# Multi-Modal Neuroimaging in Premanifest and Early Huntington’s Disease: 18 Month Longitudinal Data from the IMAGE-HD Study

**DOI:** 10.1371/journal.pone.0074131

**Published:** 2013-09-16

**Authors:** Juan F. Domínguez D, Gary F. Egan, Marcus A. Gray, Govinda R. Poudel, Andrew Churchyard, Phyllis Chua, Julie C. Stout, Nellie Georgiou-Karistianis

**Affiliations:** 1 School of Psychology and Psychiatry, Monash University, Clayton, Victoria, Australia; 2 Monash Biomedical Imaging (MBI), Monash University, Melbourne, Victoria, Australia; 3 Life Sciences Computation Centre, Victorian Life Sciences Computation Initiative (VLSCI), Melbourne, Victoria, Australia; 4 Centre for Neuroscience, University of Melbourne, Parkville, Victoria, Australia; 5 Centre for Advanced Imaging, Gehrmann Laboratory, the University of Queensland, St Lucia, Queensland, Australia; 6 Department of Neurology, Monash Medical Centre, Clayton, Victoria, Australia; Centre Hospitalier Universitaire Vaudois Lausanne - CHUV, UNIL, Switzerland

## Abstract

IMAGE-HD is an Australian based multi-modal longitudinal magnetic resonance imaging (MRI) study in premanifest and early symptomatic Huntington’s disease (pre-HD and symp-HD, respectively). In this investigation we sought to determine the sensitivity of imaging methods to detect macrostructural (volume) and microstructural (diffusivity) longitudinal change in HD. We used a 3T MRI scanner to acquire T_1_ and diffusion weighted images at baseline and 18 months in 31 pre-HD, 31 symp-HD and 29 controls. Volume was measured across the whole brain, and volume and diffusion measures were ascertained for caudate and putamen. We observed a range of significant volumetric and, for the first time, diffusion changes over 18 months in both pre-HD and symp-HD, relative to controls, detectable at the brain-wide level (volume change in grey and white matter) and in caudate and putamen (volume and diffusivity change). Importantly, longitudinal volume change in the caudate was the only measure that discriminated between groups across all stages of disease: far from diagnosis (>15 years), close to diagnosis (<15 years) and after diagnosis. Of the two diffusion metrics (mean diffusivity, MD; fractional anisotropy, FA), only longitudinal FA change was sensitive to group differences, but only after diagnosis. These findings further confirm caudate atrophy as one of the most sensitive and early biomarkers of neurodegeneration in HD. They also highlight that different tissue properties have varying schedules in their ability to discriminate between groups along disease progression and may therefore inform biomarker selection for future therapeutic interventions.

## Introduction

There is a global effort to test candidate treatments in Huntington’s disease (HD) aimed at delaying, reversing or preventing neural degeneration and the associated onset of symptoms [1-3]. To assess the efficacy of such therapeutic interventions, it is essential to be able to track *in vivo* the pathogenesis and progression of the disease with robust, quantitative clinical and neurobiological markers over relatively short time-frames [4]. Also important is integration of measures from various tissue properties, at both the macro- and micro-structural levels [5,6]. This creates an opportunity to not only identify which measure is the most sensitive in tracking change at the earliest possible time, but also which measure is most readily able to detect changes at a given stage during the disease continuum [7,8].

Macrostructural changes indicate atrophy of the striatum, cortical tissue and underlying white matter, which have been well quantified by a number of cross-sectional T_1_-weighted volumetric magnetic resonance imaging (MRI) studies during both the premanifest (pre-HD) and symptomatic (symp-HD) stages [9-16]. In addition, a number of longitudinal imaging studies (including those from PREDICT-HD and TRACK-HD) have reported robust increased rates of localized and widespread grey and white matter, cortical and subcortical atrophy in both pre-HD and symp-HD over periods as short as one year [9,10,13,16-22].

Microstructural change in HD has also been characterized, via diffusion tensor imaging (DTI) [23-31], although longitudinal investigation remains sparse. DTI measures the diffusion of water molecules in the intra- and extra-cellular space, [e.g., mean diffusivity, MD; apparent diffusion coefficient, ADC; trace of the diffusion coefficient, Trace(D)] and its directionality (fractional anisotropy, FA; parallel diffusivity, λ||; perpendicular diffusivity, λ⊥) thus providing information about tissue integrity. Cross-sectional studies have revealed differences in magnitude of white matter and striatal diffusivity in both pre-HD and symp-HD, relative to controls [23-26,32]. Weaver et al. [29] observed differences in white matter FA and λ|| throughout the brain between a mixed HD group (pre- and symp-HD) and controls over a one year period. Vandenberghe et al. [28] and Sritharan et al. [31] are the only two studies to date to investigate longitudinal diffusivity changes in subcortical grey matter structures in symp-HD [Trace(D) and MD, respectively], both reporting no significant change over time (two years and one year, respectively) in the caudate, putamen or thalamus in relatively small samples (<20 per group).

By investigating a diverse set of multi-modal neuroimaging approaches it may be possible to track the trajectory of different tissue properties in a more meaningful way. This could yield an appropriate set of biomarkers not only for HD characterization, but also for therapeutic intervention studies. In a recent study [5], our group found volume, MD and FA differed across pre-HD, symp-HD and controls; a Quadratic Discriminant Analysis revealed that the highest discriminative accuracy of pre-HD from controls was achieved in a multi-modality approach, including volume and diffusivity measures from the basal ganglia, accumbens and thalamus together with motor and neurocognitive scores. Sánchez-Castañeda et al. [6] reported atrophy and diffusion changes (MD and FA) in the basal ganglia, as well as iron accumulation (restricted to the globus pallidus) in pre-HD and symp-HD groups, relative to controls. They also found that MD was the most powerful predictor of HD development in the caudate and putamen, explaining 50% of the variance of disease progression.

IMAGE-HD is an Australian based intensive longitudinal multi-modal MRI study investigating the sensitivity of macrostructural, microstructural and functional markers at three time points: baseline, 18 and 30 months. For this investigation we report on T_1_ and diffusion weighted images from pre-HD, symp-HD and healthy controls collected at baseline and 18 months. We performed segmentation-based whole brain analyses, as well as region of interest analyses of the caudate and putamen. We selected these two structures as they exhibit the most pronounced neurodegeneration in HD and have consequently been a major focus of research [33]. We aimed to determine the efficacy with which longitudinal disease progression in HD could be tracked using macrostructural (volume) and microstructural (diffusivity) neuroimaging measures with a relatively small sample. Specifically, we expected to find increased rates of atrophy over 18 months across all volume measures in both pre-HD and symp-HD, relative to controls. With respect to diffusion measures, we predicted an increase in both FA and MD in caudate and putamen in pre-HD and symp-HD, relative to controls, as suggested by previous cross-sectional reports [6,27]. Moreover, we investigated which of these measures were the most sensitive in detecting disease related longitudinal changes, and the earliest time point during pre-HD at which change is detectable. A final objective was to evaluate the relationship between longitudinal change and disease progression from pre-HD through to symp-HD stages.

## Materials and Methods

### Ethics Statement

The study was approved by the Monash University and Melbourne Health Human Research Ethics Committees, and each participant gave written informed consent.

### Participants

One hundred and eight participants took part at baseline, 93 of whom returned for the 18 months session (4 pre-HD, 4 symp-HD and 7 controls did not take part at follow-up). Structural T_1_-weighted, and diffusion weighted scans were acquired for these participants. Scans containing image artefacts or for which measurements failed altogether were excluded, leaving a T_1_-weighted sample of 87 participants (31 pre-HD, 31 symp-HD and 27 controls) and a diffusion weighted sample of 86 participants (29 pre-HD, 29 symp-HD and 28 controls). In total, imaging data was available for 31 pre-HD, 31 symp-HD and 29 controls. CAG repeat length, established prior to enrolment in the study, ranged from 39 to 49 (mean 42.7, SD 2.1 for pre-HD; 42.7, 2.2 for symp-HD). Pre-HD and symp-HD participants were clinically assessed (by A.C. or P.C.) with the Unified Huntington’s Disease Rating Scale (UHDRS), total motor score (TMS) [34]. Consistent with criteria employed by Tabrizi et al. [15], individuals with a UHDRS TMS ≤ 5 were included in the pre-HD group and those with UHDRS TMS greater than 5 were included in the symp-HD group. Diagnostic confidence score was not taken into consideration for assignment into symp-HD group. However, the mean diagnostic confidence score for symp-HD was 2.9 (±1.24) at baseline and 3.33 (±1.30) at 18 months. At baseline, mean pre-HD estimated years to clinical diagnosis (calculated using Langbehn and colleagues’ [35] survival analysis regression equation based on CAG repeats) was 14.7 (8.0) years and mean symp-HD years since diagnosis (by means of clinical records provided by the study neurologists) was 2.1 (1.5) years. Healthy controls were matched to pre-HD participants by age, gender and estimated IQ (National Adult Reading Test 2nd edition, NART-2) [36]; IQ did not differ between groups. Demographic and clinical data are presented in Table 1. We also subdivided pre-HD into farther from (pre-HD_*far*_) and closer to (pre-HD_*close*_) diagnosis at the group median years to diagnosis (median = 14.6 years). These two groups differed from each other in terms of age, UHDRS TMS, Disease Burden Score (DBS = age×[CAG repeats-35.5] [30]; and IQ (see Table 1).

**Table 1:** Demographic and clinical baseline data across groups.

	Controls	Pre-HD_*far*_	Pre-HD_*close*_	Pre-HD_*all*_	Symp-HD
Participant No.	29	16	15	31	31
Age (years)	42.8 (12.9)	37.5 (9.5)	45 (8.5)*	41.1 (9.6)	53.1 (9.0)^***+++^
IQ estimate	118.1(10)	120.8 (10.1)	112.2 (12.4)*	116.6 (11.9)	114.5 (11.5)
UHDRS TMS	-	0.4 (0.7)	1.5 (1.5)*	0.9 (1.3)	17.6 (9.6) ^+++^
CAG repeats	-	42.2 (2.2)	42.7 (1.8)	42.7 (2.1)	42.7 (2.2)
Disease Burden Score	-	235.4 (43.5)	306.4 (37.6)^^^^^	284 (74)	370 (67) ^***^
Estimated Years to Diagnosis	-	20.5 (6.3)	10.6 (3.1) ^^^^^	14.7(8)	-
Years Since Diagnosis	-	-	-	-	2.1 (1.5)

Data represent mean(SD) at baseline and significance (superscript); IQ (NART: National Adult Reading Test 2nd Edition); UHDRS TMS: Unified Huntington’s Disease Rating Scale, total motor score (pre-HD <5; symp-HD ≥5); CAG: cytosine-adenine-guanine repeat length; Disease Burden Score = age x (CAG repeats-35.5). Significance of group differences: any HD *vs.* controls ^*^ = *p* ≤ .05; ^***^ = *p* ≤ .001; symp-HD *vs.* pre-HD: ^+++^ = *p* ≤ .001; Pre-HD_*close*_ *vs.* Pre-HD_*far*_: ^^^ ^^^^ = *p* ≤ .001.

All participants underwent a rigorous screening process prior to recruitment. Participants were all free from brain injury, neurological and/or severe diagnosed psychiatric conditions (e.g., bipolar, psychosis), other than HD. Participants remained on their normal medication regime, which included antidepressants, medications for vascular and heart conditions, anxiety/mood stabilizers and neuroleptic medications (exclusive to symp-HD). (See Methods S1 for details.) 

Consistent procedures were followed at baseline and 18-months. At each assessment, detailed demographic and clinical information was recorded, including an extensive motor, neurocognitive and neuropsychiatric battery of tests and questionnaires. T_1_ and diffusion weighted brain images were also acquired during the same session.

### Procedures

A set of pen-and-paper and computerized cognitive tests were selected based on their sensitivity in discriminating between controls, pre-HD and symp-HD groups [15,37,38]. These tests assessed visuomotor speed and attention (Symbol Digit Modalities Test, SDMT; [39]), reading speed (Stroop Word Test [40]), odour recognition (University of Pennsylvania Smell Identification Test, UPSIT [41]) and motor performance (speeded and self-paced tapping tasks [42,43]). In addition, participants completed questionnaires associated with frontal-striatal brain dysfunction, including executive function and neuropsychiatric disturbances (Frontal Systems Behavior Scale, FrSBe [44];; Schedule of Obsessions, Compulsions and Psychological Impulses, SCOPI [45];). Two additional questionnaires included the Hospital Anxiety and Depression Scale (HADS; [46]) and the Beck Depression Inventory Version II (BDI-II [47]).

Structural and diffusion MRI data acquisition protocols, spatial pre-processing and methods to derive regions of interest (ROIs) were consistent across testing sessions (see Methods S1 for details.) MRI data were acquired at 3T with standardized protocols. Rigorous quality control was carried out on all images. FMRIB’s Software Library (FSL, version 4.1.61) was used to delineate volumetric baseline and follow-up regions including whole brain (WB), grey matter (GM), white matter (WM) and cerebrospinal fluid (ventricular and intergyral). A semi-customised procedure based on SPM8 routines was used for segmentation of caudate and putamen in volumetric images. FSL tools were used for segmentation of these subcortical ROIs in diffusion weighted images. MD and FA measures were then computed for each these ROIs. Registrations and segmentations were visually inspected independently by two analysts to ensure their accuracy. Cortical and subcortical regions were derived independently for baseline and follow-up scans. In order to obtain the most precise MD measurements, we calculated change in ventricular MD across scanning sessions and controlled for this in MD analyses.

### Statistical analysis

Statistical analyses were conducted by group assignment at baseline (given that, according to UHDRS criteria, eight participants changed grouping between pre-HD and symp-HD at follow-up; see Methods S1). We assessed longitudinal change in neurocognitive measures in pre-HD, symp-HD and control groups as well as longitudinal differences between HD groups and controls. We used a random-effects model with a generalized least squared estimator (GLM), as it allows for correlations between measurements from the same participant. Outcome measures were accuracy: number correct (SDMT, Stroop), response time: intertap interval (sTAP), and precision (the inverse of intertap interval standard deviation * 1000; sPTAP, fPTAP). Age was included in all models as a covariate of no interest. Post-hot tests were corrected for multiple comparisons at *p* < 0.05.

Percent change of baseline was calculated for each volume, MD and FA measure (averaging across the left and right hemispheres for subcortical structures). Linear regressions were then used to estimate longitudinal between-group differences. Age (mean centred) was included in all models as a covariate of no interest. To guard against violations of distributional assumptions and reduce the influence of outliers, we report results from bootstrapped regressions performed on the basis of 5000 permutations. In addition, a small number of extreme scores (with a residual greater than ±3) were excluded.

We also investigated the earliest point in time before diagnosis at which group differences could be detected in any of the imaging modalities. To achieve this, we split pre-HD (at the median years to diagnosis) into pre-HD_*far*_ (far from diagnosis) and pre-HD_*close*_ (close to diagnosis) and compared them to age matched control sub-groups (mean ages[SD]: pre-HD_*far*_ = 37.5 [9.5]; and their matched control group, controls_*far*_ = 37.1 [6.3]; pre-HD_*close*_ = 45 [8.5]; controls_*close*_ = 44.7 [9.5]; pre-HD_*far*_ and controls_*far*_ were 7.5 years older than pre-HD_*close*_ and controls_*close*_ (*p* < .001); see Table 1). Sample sizes for these sub-analyses are comparable to those in the studies by Sritharan et al. [31] and Vandenberghe et al. [28]. In these sub-analyses we focused exclusively on the caudate since our original analyses revealed the largest amount of 18 month change in this structure across the imaging modalities and there is extensive research documenting caudate sensitivity in HD pathology [5,9,10,18,19,21,22,48,49].

Finally, associations between specific changes in volume and diffusion measures were assessed by means of partial correlations controlling for the effects of covariates (DBS and age). We also conducted partial correlations to investigate the relationship between change in different MR measures and clinical scores (at baseline), including DBS, disease progression and UHDRS TMS. Our definition of disease progression, a measure used previously by Sánchez-Castañeda*,* et al. [6] (which they termed HD development), is a composite of estimated years to diagnosis (in pre-HD) and years since symptom onset (in symp-HD). We also assessed the association between change in MR measures and estimated years to diagnosis and years since symptom onset separately. Associations with DBS were adjusted for age. The effects of DBS and age were removed from correlations with UHDRS TMS. Lastly, we controlled for CAG repeat length when the independent variable was disease progression. All analyses were performed with Stata 11 [50].

## Results

### Longitudinal change in neurocognitive measures

Precision in fPTAP significantly improved in pre-HD (p < .02). No significant longitudinal change in any other neurocognitive measure was observed in pre-HD or symp-HD. There was a significant longitudinal difference in sPTAP between pre-HD and controls (p < .05) and symp-HD and controls (p < .02), but this difference was driven by significantly better performance in controls between baseline and 18 months (p < .001). Performance in this measure remained unchanged in pre-HD and symp-HD. No other significant longitudinal group differences were observed.

### Longitudinal group differences in brain-wide volume change

**Figure 1:**
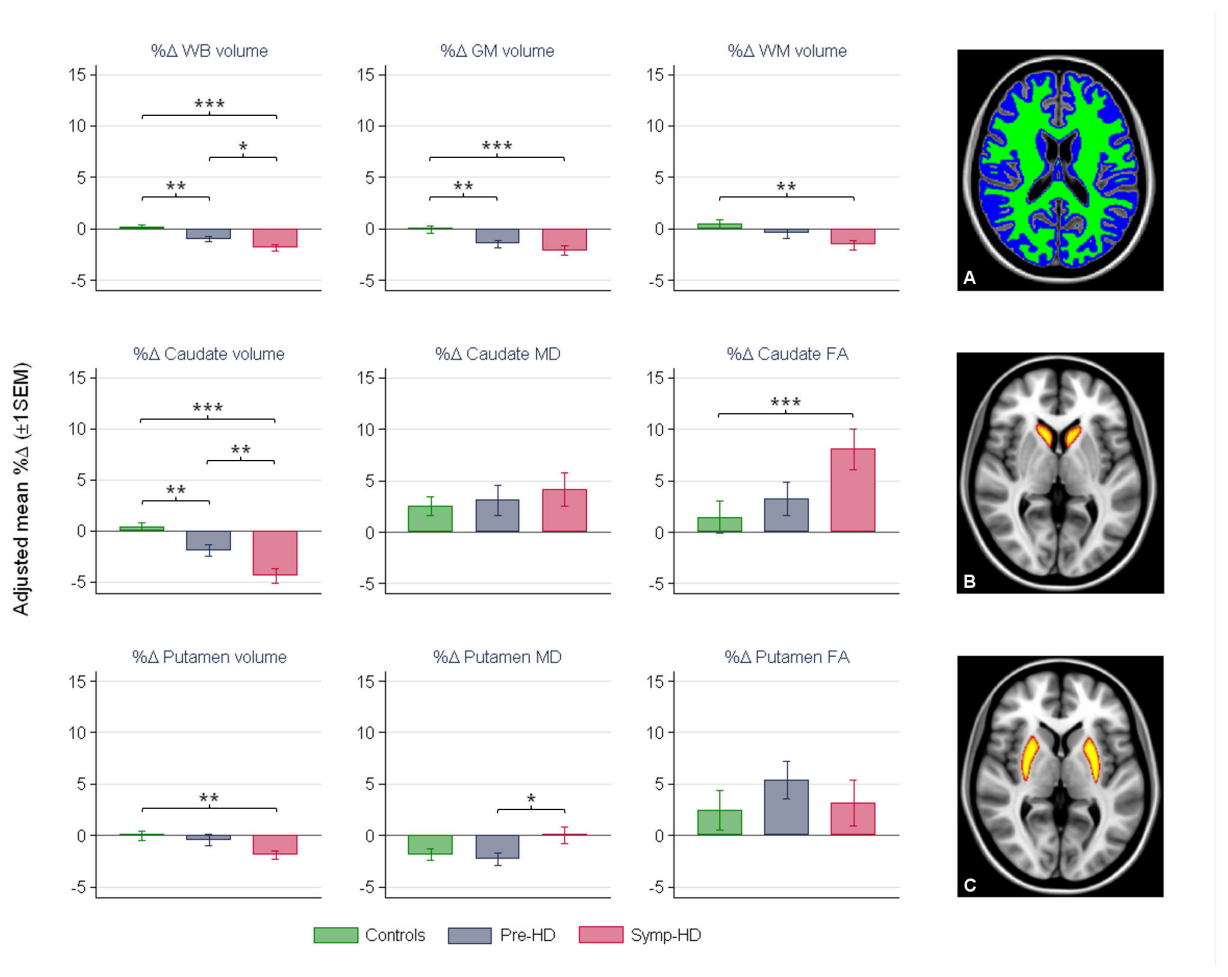
Mean percent change of baseline (%Δ) and significant group differences in volume and diffusion measures adjusted for age. Top row: brain-wide percent volume change: whole brain (WB), gray matter (GM) and white matter (WM); middle and bottom rows: subcortical percent volume, MD and FA change (caudate and putamen, respectively). Stars indicate significant between-group differences: * *p* ≤ .05; ** *p* ≤ .01; *** *p* ≤ .001. Cortical and subcortical segmentations overlaid on MNI T_1_ 1 mm^3^ standard brain are provided in right column: A) grey and white matter depicted in blue and green, respectively; B) caudate and C) putamen are represented in red-yellow.

Pre-HD and symp-HD groups demonstrated significantly higher 18-month WB and GM rates of atrophy, compared with controls. Symp-HD also exhibited significantly higher longitudinal rates of atrophy in WB compared with pre-HD, and in WM relative to controls (see Figure 1; see Tables S1 and S2 for a full inventory of longitudinal within- and between-group regression coefficients and their statistical significance; see Table S4 for annualized rates of change across volume, FA and MD measures). While no difference was observed between longitudinal rate of change in WM volume between pre-HD and controls, there was a significant difference of 2.03% (*p* = .01) in WM volume loss when comparing pre-HD_*close*_ to their age-matched controls.

### Longitudinal group differences in subcortical volume change

The rate of atrophy over 18 months in the caudate was significantly higher in pre-HD and symp-HD, compared with controls, as well as in pre-HD, compared to symp-HD (see Figure 1 and Table 2). In addition, while symp-HD showed significantly higher longitudinal rates of atrophy in the putamen compared with controls, there was no difference between pre-HD and controls. The difference between symp-HD and pre-HD in the rate of putamen volume loss exhibited a trend toward significance (see Figure 1; see Tables S1 and S2 for a full inventory of longitudinal within- and between-group regression coefficients and their statistical significance).

**Table 2:** Within- and between-group longitudinal rate of change in caudate volume, FA and MD in controls, pre-HD and symp-HD.

	N	Volume	MD	FA
Within-groups %Δ				
Controls_*far*_	22	0.38 (0.44)	0.56 (1.08)	0.004 (1.89)
Controls_*close*_	18	0.41 (0.56)	2.05 (1.29)	1.29 (2.15)
Controls_*all*_	29	0.39 (0.42)	2.52 (0.96)^**^	1.45 (1.58)
Pre-HD_*far*_	16	-1.75 (0.62)^**^	-0.58 (1.30)	3.61 (2.50)
Pre-HD_*close*_	15	-2.19 (0.91)^*^	5.62 (1.97)^**^	1.90 (2.28)
Pre-HD_*all*_	31	-1.92 (0.58)^***^	3.12 (1.47)^*^	3.21 (1.63)^*^
Symp-HD_*all*_	31	-4.43 (0.71)^***^	4.17 (1.62)^**^	8.08 (2.00)^***^
Between-groups %Δ				
(Pre-HD *vs.* Controls)_*far*_		-2.13 (0.75)^**^	-1.14 (1.71)	3.61 (3.16)
(Pre-HD *vs.* Controls)_*close*_		-2.61 (1.06)^**^	3.56 (2.38)	0.62 (3.15)
(Pre-HD *vs.* Controls)_*all*_		-2.31 (0.69)^**^	0.60 (1.74)	1.76 (2.29)
(Symp-HD *vs.* Controls)_*all*_		-4.82 (0.87)^***^	1.64 (1.96)	6.63 (2.53)^**^
(Symp-HD *vs.* Pre-HD)_*all*_		-2.51 (0.96)^**^	1.04 (2.20)	4.86 (2.57)^+^

Within-groups % Δ (percent change of baseline): Data represent mean (SE) rates of change (adjusted for age at baseline). Controls far and close are subsamples of the control group matching in age the respective pre-HD_*far*_ and pre-HD_*close*_ groups. Subscript all indicates full samples. Stars (*) indicate *p* value for within-group effect on rate of change: ^*^ *p* ≤ .05; ^**^ *p* ≤ .01; ^***^ *p* ≤ .001. Between-groups % Δ: Data represent differences in the rate of change between groups (SE). Stars (*) and plus sign (+) indicate *p* value for the between-group differences: ^*^ *p* ≤ .05; ^**^ *p* ≤ .01; ^***^ *p* ≤ .001; ^+^ *p* = .058.

### Longitudinal group differences in subcortical diffusivity change

While there was a significant within-group longitudinal increase in caudate MD across all groups, no significant longitudinal between-group difference was observed. Pre-HD exhibited a significant longitudinal reduction in putamen MD, relative to symp-HD. The longitudinal increase in caudate FA was significantly higher in symp-HD (8.1%), relative to controls (1.5%). Although the pre-HD group showed a trend in the same direction, the difference between pre-HD and symp-HD exhibited only a trend toward significance (*p* = .058), and the difference between pre-HD and controls was not significant (see Figure 1 and Table 2; see Tables S1 and S2 for a full inventory of longitudinal within- and between-group regression coefficients and their statistical significance).

While longitudinal change in caudate volume and FA discriminated symp-HD from controls, neither measure was better at detecting a difference between these groups. The difference between symp-HD and controls in FA change (6.6%) was higher than the difference in volume change (4.82%), but not significantly so, as revealed by a direct post-estimation comparison.

### Longitudinal change in caudate volume, MD and FA in pre-HD_far_ and pre-HD_close_

We observed a significant (p < .01) group difference in rate of caudate atrophy (2.8%) for pre-HD_*far*_, compared with controls_*far*_ (see Table 2). The difference in rate of atrophy between pre-HD_*close*_ and controls_*close*_ (2.6%) was also significant (p < .01). No significant group differences were observed in MD or FA.

### MR and clinical correlations

**Figure 2:**
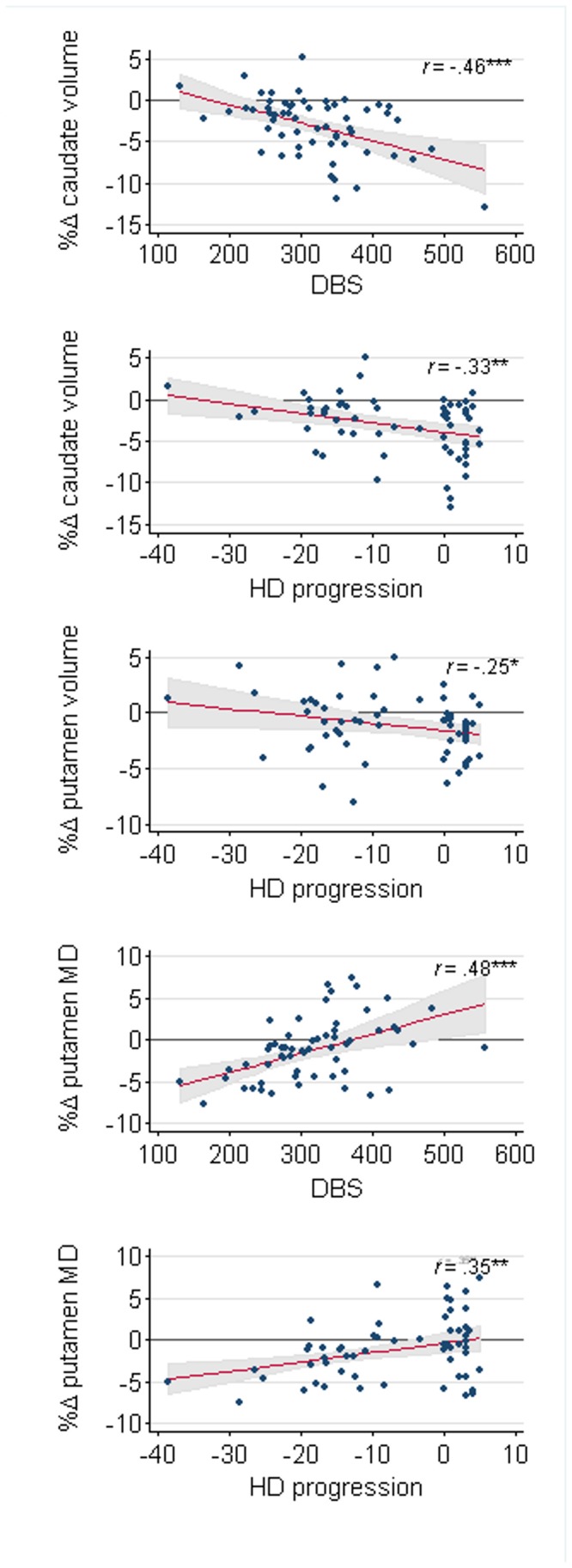
Significant correlations between percent change of baseline (%Δ) in caudate and putamen MR measures and clinical scores. Plots show fitted lines (and 95% CI, shaded area) adjusted for covariates. DBS: Disease Burden Score; HD progression is a composite of estimated years to clinical diagnosis (expressed in negative numbers as count to 0) and years since diagnosis (count from 0).

After controlling for covariates, we found significant negative associations between rate of change in the caudate for volume and FA (*r* = -.35, *p* < .05) and the putamen for volume and MD (*r* = -.32, *p* < .01), and for FA and MD (*r* = -.36, *p* < .01). Regarding clinical measures, increased rate of WB volume loss negatively correlated with disease progression. Percent change in caudate volume negatively correlated with DBS, HD progression and UHDRS TMS. Putamen atrophy was negatively correlated with HD progression; and putamen rate of change in MD was associated positively with DBS, HD progression, pre-HD progression and UHDRS TMS (See Figure 2 and Table S3).

## Discussion

In the present study we report a range of significant volumetric and diffusion changes over an 18 month period in both pre-HD and symp-HD, relative to controls, detectable at both the brain-wide level and in the caudate and putamen. Our findings also characterise the differential patterns of rate of change across multiple measures and at different stages of the disease continuum and provide further insight into early neuropathological changes in HD. Below we summarise these results and their significance.

### Longitudinal group differences in the caudate

Consistent with previous reports [9,10,18,19,21,22,48,49], we observed higher rates of caudate atrophy over 18 months in pre-HD and symp-HD, compared with controls, and in symp-HD, compared to pre-HD. In addition, we report for the first time longitudinal FA change in HD. In particular, caudate FA increased over 18 months by 8% in symp-HD, which was significantly greater than the increase observed in controls (1.5%). Caudate MD increased in all groups over the same period (seemingly higher in symp-HD, followed by pre-HD then controls), but no significant differences were observed on this measure between the groups. We also observed a difference in the rate of change in caudate volume between pre-HD_*far*_ and controls_*far*_, and pre-HD_*close*_ and controls_*close*_. There were no diffusion related differences in the caudate between pre-HD_*far*_ or pre-HD_*close*_ and controls. However, we observed a marked longitudinal increase in MD in pre-HD_*close*_, sustained after diagnosis, suggesting a likely change in the magnitude of diffusion with the approach of symptom onset.

The caudate findings highlight the complexity of longitudinal change in multimodal MR measures by their varying schedules in discriminating between groups along the disease continuum. Specifically, longitudinal volume change in the caudate was the only measure that discriminated between groups across all stages of disease: far from diagnosis (>15 years), close to diagnosis (<15 years) and after diagnosis. Of the two diffusion metrics, only longitudinal FA change was sensitive to group differences but only after diagnosis. Caudate MD exhibited a trend toward increasing across far, close and after symptom onset, which suggests that MD may become sensitive to group differences at more advanced stages of disease. These findings further confirm caudate atrophy as one of the most sensitive measures of neurodegeneration in HD, capable of detecting group differences at >15 years from diagnosis.

### Longitudinal group differences in the putamen

In line with previous findings, we observed significant longitudinal putamen volume decrease in symp-HD, compared with controls; however, and contrary to findings by a number of groups, there was no difference between pre-HD and controls [17,18,21,49]. This discrepancy may be due to characteristics of our sample. However, our results may also have been affected by segmentation difficulties intrinsic to the putamen (even after stringent quality control) and/or differences in the tools used in delineating the corresponding masks. In addition, greater variability in the rate of volume loss in this structure has been previously reported [21].

Putamen FA exhibited a significant longitudinal increase in pre-HD; however, there was no statistically significant difference in the rate of change between groups. While this study is the first to demonstrate *longitudinal* change in putamen FA, our cross-sectional data [5] is consistent with previous results [6,25,27] which indicate that FA increases with disease progression likely due to selective fibre degeneration. Putamen MD showed no longitudinal change in symp-HD, although there was a significant longitudinal decrease in both pre-HD and controls. The lack of longitudinal change in symp-HD is also in accord with previous studies [28,31].

Of interest are the divergent results for MD measures across caudate and putamen. These structures have the same phylogenesis, share a predominance of medium-size spiny neurons, and have similar patterns of connectivity and dendritic arborisation, so one might expect a similar pattern of deterioration [51,52]. However, while caudate MD significantly increased longitudinally in all groups, putamen MD significantly decreased in controls and pre-HD, and no change was observed in the symp-HD group.

In pre-HD (and controls), we observed an 18 month MD decrease in putamen. This is surprising, especially when considering that, cross-sectionally, MD was highest in symp-HD, followed by pre-HD then controls (a finding consistent with previous reports [5,6,23,53]. There are a number of neuropathological and age-related processes that may possibly account for this pattern of longitudinal change. These include for example increased oligodendroglial numbers present in HD developmentally [30,52,54], homeostatic attempts to remyelinate [55], and iron accumulation. The latter occurs naturally with the aging process [56-58], but also increases with greater numbers of oligodendroglia and remyelination in HD [6,55,59]. Larger numbers of oligodendroglia, remyelination and iron accumulation may contribute to real and apparent decreases in diffusivity, the former two by increasing barriers to water diffusivity and the latter by introducing magnetic susceptibility effects. These processes may combine to produce a pattern of short-term striatal MD decreases against the backdrop of a long-term trend toward higher diffusivity (e.g., [30] reported reduced caudate MD in pre-HD far from diagnosis relative to controls). In the putamen, this pattern may be amplified due to the combination of two factors: the putamen has a higher iron concentration than the caudate [56,57]; and second, the rate of iron deposition keeps increasing throughout the lifespan in this structure whereas it plateaus in the caudate around the third decade of life [57,58]. Inter-individual fluctuations in iron deposition, that occur with increased age [58], may also play a role. The lesser putamen MD reduction in controls may be accounted for by similar processes occurring in the absence of pathology. However, it is important to highlight that the present results may have been affected by difficulties in the segmentation procedures. Validation is therefore required from further studies.

### Brain-wide longitudinal group differences

Symp-HD demonstrated the largest longitudinal rates of atrophy in WB, GM and WM, which were significantly different from controls in all cases, and in pre-HD for WB only. Pre-HD also showed larger rates of atrophy, compared with controls, in WB and GM. Longitudinal atrophy in WB also discriminated symp-HD from pre-HD. These results are largely in agreement with previous studies [13,17,18,21,49]; however, pre-HD did not exhibit a significantly larger longitudinal rate of loss in WM. While this is consistent with our cross-sectional findings, where no WM difference was observed between pre-HD and controls [5], both PREDICT-HD [49] and TRACK-HD [21] have reported cross-sectional and longitudinal differences between pre-HD (far and close to onset) and controls. The discrepancy may be in part due to different methods (voxel-based morphometry *vs.* whole volume segmentation), sample sizes, and disease stages. When restricting our analysis to pre-HD_*close*_, however, we observed that the rate of WM atrophy was significantly higher than in controls, suggesting that by 10.6 years prior to diagnosis a sample of n = 17 per group may be sufficient to detect significant WM atrophy.

### MR and clinical associations

We found for the first time evidence that changes in volume and diffusion metrics are interrelated, as indicated by the significant correlations between longitudinal change in caudate volume and longitudinal change in caudate FA; and between longitudinal change in putamen MD and longitudinal change in putamen volume and FA. In line with previous reports [18,21,49], we observed significant negative associations between rate of longitudinal change in a number of volume measures and several clinical measures: caudate atrophy was correlated with DBS, HD progression and UHDRS TMS; and WB and putamen atrophy were correlated with HD progression. Regarding the diffusion metrics, positive correlations were found between change in putamen MD and DBS, HD progression, pre-HD progression and UHDRS TMS. These positive putaminal correlations point to an on-going reversal of the decrease in diffusivity in pre-HD, which illustrates the longitudinal MD reduction prior to diagnosis and is consistent with a return to a long-term trend of increased diffusivity. These findings collectively provide further support that longitudinal neuropathological changes in caudate, putamen and WB volume and in putamen MD are related to functional measures of disease severity.

The clinical metrics used in the present study measure closely related constructs (i.e., clinical severity, disease progression). However, they can be expected to have different variability and may therefore differ in their sensitivity to changes in MR measures. This is particularly the case when directly comparing DBS and HD progression, which are used to evaluate the clinical relevance of changes in MR measures across the entire spectrum of the disease. Care should however be taken when interpreting correlations comprising the HD progression variable. This variable treats as a continuum two separate measures: estimated years to onset and years since diagnosis with potentially different trajectories as well as levels of uncertainty and variability. This makes it necessary to consider these stages separately. In addition, different clinical measures have been used across published studies and comparing them side by side may provide insights as to which one is potentially more sensitive for any given structure and modality. For example, DBS was more strongly correlated with change in caudate and putamen volume than HD progression. However, only HD progression was found to be associated with whole brain volume.

### Longitudinal change in neurocognitive measures

We found no longitudinal deterioration in neurocognitive measures in either pre-HD or symp-HD. This is in agreement with previous findings which show that clinical scores, and performance on neurocognitive tests, do not deteriorate to the same extent in HD as cortical and subcortical measures of volume loss [22], likely due to compensatory neural processes to maintain function [60]. The absence of cognitive decline also highlights the superior sensitivity of MR measures in detecting differences relating to neuropathology.

### Variability across multi-modal measures

One factor that is likely to influence the sensitivity of imaging measures is their high degree of variability. In the caudate nucleus, although diffusivity measures exhibited larger longitudinal rates of change, they were more variable than volume (FA having the largest variability, followed by MD, then by volume). Therefore, there may be a trade-off between smaller variability in longitudinal volume change and higher effect sizes in longitudinal diffusivity change. This greater variability in longitudinal diffusivity may have contributed to the non-significant longitudinal change between groups in caudate MD (two previous studies with smaller samples failed to report longitudinal within-group change in the magnitude of diffusion in symp-HD [28,31]). Our results therefore indicate that change in caudate volume is a more sensitive measure of longitudinal group differences compared with the diffusivity measures.

## Conclusions

This study sought to determine the sensitivity of macrostructural and microstructural change longitudinally in HD at different stages of the disease. Our findings highlight the importance of understanding how different tissue properties can change over time, whether such changes progress at the same rate, which commence earlier, and whether change in different types of tissue is comparatively more sensitive at different stages. Our findings also lend further support to previous studies documenting longitudinal volumetric sensitivity in various structures (WB, GM, WM), which we found to be associated with clinically relevant symptoms. Most importantly, our results show for the first time that while both structural and microstructural measures of longitudinal change in the caudate are sensitive to disease progression, volume is more sensitive than MD and FA to longitudinal group differences; it is the only metric sensitive to such differences before diagnosis; it is capable to detect differences very early (i.e., >15 years prior to symptom onset); and it is highly relevant clinically. These findings illustrate the sensitivity of multi-modal longitudinal imaging at detecting and tracking short-term change along the progression of the disease and may inform biomarker selection for future therapeutic interventions. 

## Supporting Information

Figure S1 **Automated identification of subcortical regions of interest in diffusion weighted images across participant groups.** Provided are representations of one control and one symp-HD participant for illustrative purposes. A) Raw FA and T1 weighted images. B) T1 weighted images were first linearly and then non-linearly registered to the corresponding FA images. C) Segmentation of subcortical structures of interest (caudate and putamen) was performed on the non-linearly registered T1 image; these structures were then boundary corrected (eroded boundary in yellow). Eroded masks for the caudate (red) and putamen (blue) are displayed over FA map.
(TIF)

Figure S2 **Comparison between linear and non-linear registration of T1 and diffusion images.**
(TIF)

Methods S1 (DOCX)

Table S1 **Within-groups longitudinal results across volume, MD and FA.**
(DOCX)

Table S2 **Between-groups longitudinal results across volume, MD and FA.**
(DOCX)

Table S3 **Associations between MR percent change measures and clinical scores.**
(DOCX)

Table S4 **Annualized rates of change across volume, FA and MD measures.**
(DOCX)
