# Supplementary figures and images for: Multi-Modal Neuroimaging in Premanifest and Early Huntington’s Disease: 18 Month Longitudinal Data from the IMAGE-HD Study
Source: PLoS One. 2013 Sep 16;8(9):e74131. doi: 10.1371/journal.pone.0074131 (PMC3774648; doi:10.1371/journal.pone.0074131)

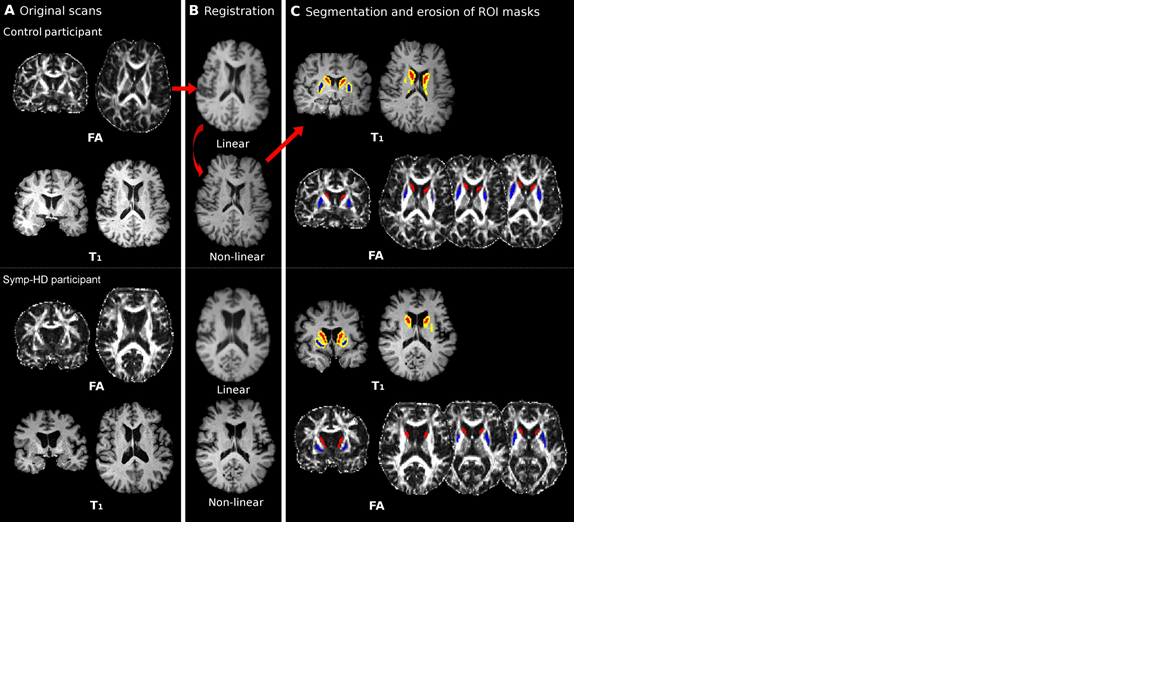

Supplement: Figure S1 — Automated identification of subcortical regions of interest in diffusion weighted images across participant groups. Provided are representations of one control and one symp-HD participant for illustrative purposes. A) Raw FA and T1 weighted images. B) T1 weighted images were first linearly and then non-linearly registered to the corresponding FA images. C) Segmentation of subcortical structures of interest (caudate and putamen) was performed on the non-linearly registered T1 image; these structures were then boundary corrected (eroded boundary in yellow). Eroded masks for the caudate (red) and putamen (blue) are displayed over FA map. (TIF) [file pone.0074131.s001.tif]

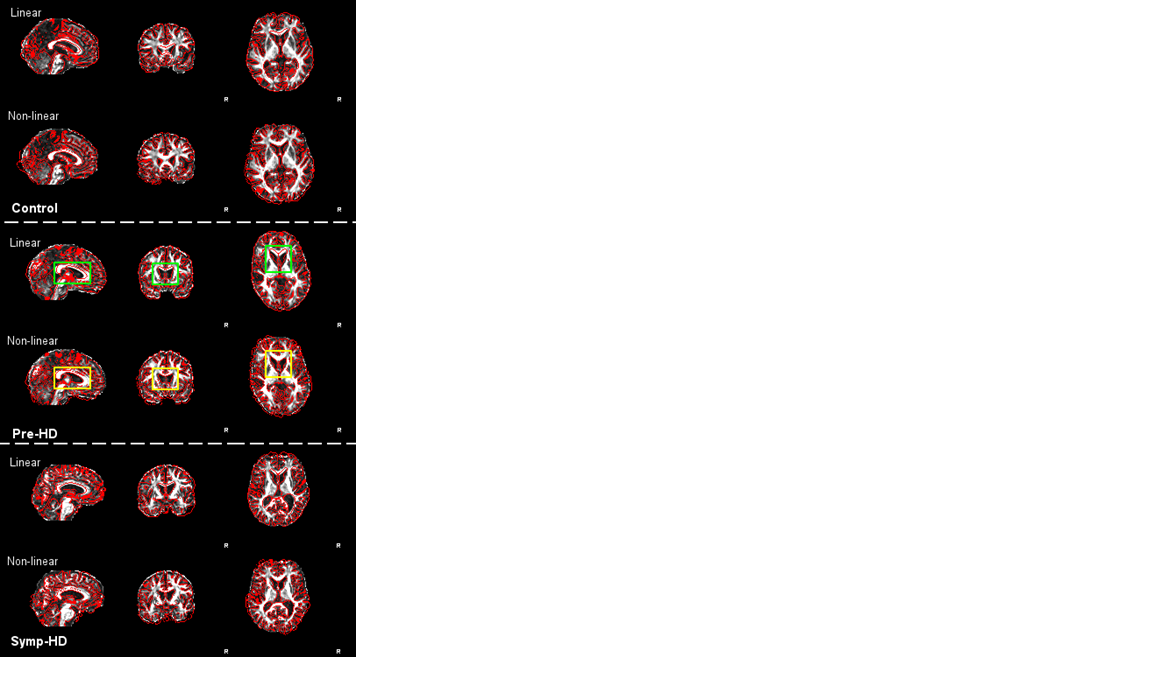

Supplement: Figure S2 — Comparison between linear and non-linear registration of T1 and diffusion images. (TIF) [file pone.0074131.s002.tif]
