# Supplementary material for: Multi-Modal Neuroimaging in Premanifest and Early Huntington’s Disease: 18 Month Longitudinal Data from the IMAGE-HD Study
Source: PLoS One. 2013 Sep 16;8(9):e74131. doi: 10.1371/journal.pone.0074131 (PMC3774648; doi:10.1371/journal.pone.0074131)
Supplement: Methods S1 — (DOCX) [file pone.0074131.s003.docx]

**Methods S1**

***Clinical characteristics of IMAGE-HD cohort at baseline***

HD gene carriers were analysed according to group assignment at baseline. However, there were eight participants whose status as pre-HD and symp-HD following UHDRS scoring changed across testing sessions. Six participants diagnosed as pre-HD at baseline obtained a score greater than five when assessed 18 months later (range 7 to 13). A further two participants diagnosed as symp-HD at the first testing session were scored 5 and 2 the second time around.

***Medication regime***

Controls reported taking medications for vascular and heart conditions (baseline n= 3, 18 months = 3), selective serotonin reuptake inhibitor (SSRI) antidepressants (1, 0) and serotonin-norepinephrine reuptake inhibitor (SNRI) antidepressants (0, 1). Pre-HD participants reported taking medications for vascular and heart conditions (1, 2), SSRI (4, 6) and noradrenergic and specific serotonergic antidepressants (NaSSA) (1, 1). Symp-HD participants reported taking medications for vascular and heart conditions (3, 6), SSRI (9, 12), SNRI (1, 2) and NASSA (1, 2), as well as anxiety/mood stabilizers (6, 3), anticonvulsants (0, 1) and neuroleptic medications (8, 9).

***Procedures***

MRI scanning (Magnetom Trio 3 Tesla, Siemens AG, Erlangen, Germany) with a 32-channel head coil was conducted at the Murdoch Children’s Research Institute (Royal Children’s Hospital, Victoria, Australia). High-resolution T_1_-weighted images (192 slices, slice thickness of 0.9 mm, 0.8 mm x 0.8 mm in-plane resolution, 320 x 320 field of view, TR = 1900 ms, TE = 2.6 ms, flip angle = 9°) and diffusion weighted scans were acquired (double spin echo EPI sequence, TR = 8200 ms, TE = 89ms, flip angle = 90˚, 64 contiguous slices, 2mm isotropic voxels, acquisition matrix 128x128, 60 direction encoding gradients, b = 1200 s/mm^2^, ten b0 images).

The spatial normalization and segmentation procedures are the same as in our previous cross-sectional multi-modal study [[1](#_ENREF_1)], except for minor adjustments to the procedure used to define structural ROIs to improve sensitivity. In the context of brain-wide volume measurements, all non-brain tissue was removed from the T_1_-weighted images of each participant with BET (Brain Extraction Tool, FMRIB’s Software Library–FSL, 4.1.61; [[2](#_ENREF_2)]. Brain extraction for five participants required correction by hand. FAST (FSL’s Automated Segmentation Tool) was used to segment each participant’s T_1_-weighted image, whilst correcting for spatial intensity variations, into tissue type: grey matter (GM), white matter (WM) and (ventricular and intergyral) cerebrospinal fluid (CSF). Volume measurements were then estimated from these segmentations. Additionally, we calculated whole brain volume (WB, as the sum of GM and WM volumes) and total intracranial volume (as the sum of WB and CSF volumes).

Automated segmentation of caudate and putamen was made significantly more difficult by neural degeneration in HD. We tested a variety of methods and brain imaging tools, before adopting a semi-customised procedure based on SPM8 routines that yielded the most reliable results. This procedure was applied consistently at baseline and 18 months (see Georgiou-Karistianis, *et al.* [[1](#_ENREF_1)] for figure schematizing the procedure). This allowed an automated segmentation of subcortical structures with the precision required for accurate volume estimation. The procedure utilised standardised subcortical masks from the Harvard Oxford Atlas, which were optimised by hand to best fit the standard (MNI) whole brain template. Our procedure placed particular emphasis on accurate identification of peri-ventricular CSF boundaries, as they are highly sensitive to HD status and progression. Inaccuracies in identifying these boundaries have been reported in fully automated segmentation methods used for identifying subcortical structures [[3](#_ENREF_3),[4](#_ENREF_4)], especially in symp-HD participants. Individual structural scans were co-registered to MNI space, and normalisation parameters derived using SPM8 segment routine, with permissive warping regularisation (sampling distance of 1mm; regularisation factor of 0.01; discrete cosine transform warp frequency cut off of 22). Individual normalised scans were then masked, with the caudate mask allowed to extend into CSF space. Caudate and putamen masks were individually eroded using SPM8’s probabilistic spatial estimate of CSF and white matter from each scan. Registrations and segmentations were visually inspected independently by two analysts, who were satisfied that this method produced highly accurate results. Volume estimates were then generated by warping the resulting masks back into subject-space.

In order to avoid partial volume effects, which may result from larger voxel sizes in diffusion weighted scans, and misalignment between structural and diffusion images, we utilised independent ROI identification in diffusion images. Diffusion weighted images (DWI) were brain extracted (BET) and corrected for movement and eddy current induced distortions via affine registration to a reference b0 volume (FMRIB’s Diffusion Toolbox, FDT). Calculation of diffusion tensors and principal eigenvectors allowed derivation of voxel-wise MD and FA maps. MD and FA measures were then computed for each subcortical ROI using structure specific masks. First, each participant’s brain-extracted T_1_ image was non-linearly registered to its corresponding FA map (FMRIB's Nonlinear Image Registration Tool, FNIRT). Segmentation of subcortical structures of interest was performed on the non-linearly registered T_1_ image with FIRST (FSL automated segmentation and registration tool that uses Bayesian shape and appearance models). We applied the default boundary correction methods included in FIRST segmentations [[3](#_ENREF_3)]: caudate boundaries were eroded using intensity based Gaussian mixture-models identifying CSF, GM and WM and applied via Markov Random Field. Putamen erosions were achieved by fitting surfaces to the manual models after erosion by 0.5 mm from the outer boundary. The masks generated were then binarised and used to obtain FA and MD measures from the respective DTI maps (see Figure S1 below for schematic representation of the procedure). The analysts were satisfied that the masks did not overlap with CSF or WM, were well aligned with CSF and WM boundaries, and covered the extent of the structures. In order to obtain the most precise measurements, we calculated change in ventricular MD across scanning sessions and controlled for this in MD analyses.

Non-linear registration between T_1_ and DWI images was used because linear registration alone was in practice unsatisfactory. In particular, linear registration performed very badly in peri-ventricular subcortical structures, which are the focus of interest in our study. Figure S2 figure contains FA maps overlayed with edge outlines of T_1_ images (in red) that have been linearly or non-linearly registered to the FA maps. We provide one example from each group. In every case, the peri-ventricular structures are miss-aligned to the linearly registered image (highlighted in green squares in pre-HD participant). In contrast, the outcome of non-linear registration is a very precise alignment of these regions (highlighted in yellow squares in pre-HD participant).

As Georgiou-Karistianis *et al.* [[5](#_ENREF_5)] have highlighted in a recent review on the role of neuroimaging in future clinical trials in HD, conclusive recommendations for trial design are limited by discrepancies between published studies. In this context, it is important to highlight that our methods to segment cortical and subcortical ROIs can be replicated in a standardised manner without manual intervention. The standardised subcortical masks from the Harvard Oxford Atlas, which were optimised by hand to best fit the standard (MNI) whole brain template for segmentation of structural caudate and putamen ROIs, are available upon request.

***Statistical analysis***

Bootstrapping is recommended when the theoretical distribution of a statistic is complicated or unknown [[6](#_ENREF_6)]. The theoretical distribution of longitudinal change in brain volume and diffusion measures has not been well characterised and may be complicated given the potentially dynamic nature of neurodegeneration. Rather than making assumptions regarding these distributions, we decided it would be more prudent to report results from bootstrapped regressions to guard against potential violations of distributional assumptions.

Statistically significant results in this paper are not corrected for multiple comparisons given our interest in brain areas specified in advance, *i.e.*, WB, GM, WM, caudate and putamen, which have, in addition, been implicated in HD in previous neuroimaging studies (see review in introduction). Furthermore, the large number of individual tests would make it excessively stringent to correct for a global family-wise error rate of 5%. Therefore, following Tabrizi *et al.* [[7](#_ENREF_7)], who had a sample nearly four times larger as in the present paper, we report the full set of longitudinal results (see Tables S1, S2 and S3) to provide the relevant context from which readers can assess for themselves how meaningful the statistically significant tests in this paper are.

***Partial correlations***

We calculated partial Pearson correlations between brain structures and clinical measures (see Table S3). We controlled for Disease Burden Score (DBS), [[8](#_ENREF_8)] and age at baseline [[9](#_ENREF_9)]. DBS indicates the accumulated effect of mutant huntingtin at any age [[8](#_ENREF_8)]. Partialling out DBS helps control for effects of clinical severity that are additional to a common propensity towards HD disease progression. However, correcting for DBS only

**References**

1. Georgiou-Karistianis N, Gray MA, Domínguez D JF, Dymowski AR, Bohanna I, et al. (2013) Automated differentiation of pre-diagnosis Huntington's disease from healthy control individuals based on quadratic discriminant analysis of the basal ganglia: The IMAGE-HD study. Neurobiol Dis 51: 82-92.

2. Smith AB, Taylor E, Brammer M, Rubia K (2004) Neural correlates of switching set as measured in fast, event-related functional magnetic resonance imaging. Hum Brain Mapp 21: 247-256.

3. Nelson HE, Willison J, Owen AM (1992) National Adult Reading Test, 2nd Edition. Int J Geriatr Psychiatry 7: 533.

4. Morey RA, Selgrade ES, Wagner HR, Huettel SA, Wang L, et al. (2010) Scan-rescan reliability of subcortical brain volumes derived from automated segmentation. Hum Brain Mapp 31: 1751-1762.

5. Georgiou-Karistianis N, Scahill R, Tabrizi SJ, Squitieri F, Aylward E (2013) Magnetic resonance imaging in Huntington’s disease and recommendations for its potential use in clinical trials. Neurosci Biobehav Rev: In press.

6. Adèr HJ, Mellenbergh GJ, Hand DJ (2008) Advising on research methods: A consultant's companion. Huizen: Johannes van Kessel Publishing.

7. Tabrizi SJ, Scahill RI, Durr A, Roos RA, Leavitt BR, et al. (2011) Biological and clinical changes in premanifest and early stage Huntington's disease in the TRACK-HD study: the 12-month longitudinal analysis. Lancet Neurol 10: 31-42.

8. Penney JB, Vonsattel J-P, Macdonald ME, Gusella JF, Myers RH (1997) CAG repeat number governs the development rate of pathology in Huntington's disease. Ann Neurol 41: 689-692.

9. Tabrizi SJ, Reilmann R, Roos RA, Durr A, Leavitt B, et al. (2012) Potential endpoints for clinical trials in premanifest and early Huntington's disease in the TRACK-HD study: analysis of 24 month observational data. Lancet Neurol 11: 42-53.
