# Supplementary material for: Multi-Modal Neuroimaging in Premanifest and Early Huntington’s Disease: 18 Month Longitudinal Data from the IMAGE-HD Study
Source: PLoS One. 2013 Sep 16;8(9):e74131. doi: 10.1371/journal.pone.0074131 (PMC3774648; doi:10.1371/journal.pone.0074131)
Supplement: Table S1 — Within-groups longitudinal results across volume, MD and FA. (DOCX) [file pone.0074131.s004.docx]

| **Table S1. Within-groups longitudinal results across volume, MD and FA.** | | | |
| --- | --- | --- | --- |
| Volume | Controls %Δ | Pre-HD %Δ | Symp-HD %Δ |
| Whole brain | 0.16(0.22) | -0.99(0.24)^***^ | -1.81(0.31)^***^ |
| Grey Matter | -0.07(0.37) | -1.48(0.38)^***^ | -2.10(0.43)^***^ |
| WM | 0.50(0.38) | -0.44(0.46) | -1.57(0.46)^***^ |
| CSF | 2.24(0.61)^***^ | 2.68(0.79)^***^ | 3.04(0.87)^***^ |
| Caudate | 0.39(0.42) | -1.92(0.58)^***^ | -4.43(0.71)^***^ |
| Putamen | -0.002(-0.47) | -0.46(0.57) | -1.87(0.43)^***^ |
| MD |  |  |  |
| Caudate | 2.52(0.96)^**^ | 3.12(1.47)^*^ | 4.17(1.62)^**^ |
| Putamen | -1.86(0.58)^***^ | -2.29(0.59)^***^ | .05(0.79) |
| FA |  |  |  |
| Caudate | 1.45(1.58) | 3.21(1.63)^*^ | 8.08(2.00)^***^ |
| Putamen | 2.45(1.92) | 5.39(1.82)^**^ | 3.18(2.21) |
| Longitudinal change within groups. Data are adjusted % mean change of baseline (±SE) within-groups and significance (superscript): ^*^ *p≤ .*05; ^**^ *p≤ .*01; ^***^ *p≤ .*001. | | | |
