# Supplementary material for: Multi-Modal Neuroimaging in Premanifest and Early Huntington’s Disease: 18 Month Longitudinal Data from the IMAGE-HD Study
Source: PLoS One. 2013 Sep 16;8(9):e74131. doi: 10.1371/journal.pone.0074131 (PMC3774648; doi:10.1371/journal.pone.0074131)
Supplement: Table S2 — Between-groups longitudinal results across volume, MD and FA. (DOCX) [file pone.0074131.s005.docx]

| **Table S2. Between-groups longitudinal results across volume, MD and FA.** | | | |
| --- | --- | --- | --- |
| Volume | Pre-HD *vs.* Controls  %Δ | Symp-HD *vs.* Controls %Δ | Symp-HD *vs.* Pre-HD  %Δ |
| Whole brain | -1.16(0.32)^***^ | -1.98(0.38)^***^ | -0.82(0.38)^*^ |
| Grey Matter | -1.41(0.51)^**^ | -2.03(0.60)^***^ | -0.62(0.58) |
| WM | -0.94(0.56) | -2.07(0.65)^**^ | -1.13(0.66) |
| CSF | 0.45(1.00) | 0.80(1.07) | 0.35(1.15) |
| Caudate | -2.31(0.69)^***^ | -4.82(0.87)^***^ | -2.51(0.96)^**^ |
| Putamen | -0.46(0.74) | -1.86(0.63)^**^ | -1.40(0.74) ^.058^ |
| MD |  |  |  |
| Caudate | 0.60(1.74) | 1.64(1.96) | 1.04(2.20) |
| Putamen | -0.43(0.81) | 1.91(1.00) ^.057^ | 2.33(1.04)^*^ |
| FA |  |  |  |
| Caudate | 1.76(2.29) | 6.63(2.53)^**^ | 4.86(2.57)^.058^ |
| Putamen | 2.94(2.55) | 0.73(3.07) | -2.21(3.01) |
| Longitudinal change between groups. Data are adjusted differences in % mean change (±SE) between-groups and significance (superscript): ^*^ *p≤ .*05; ^**^ *p≤ .*01; ^***^ *p≤ .*001; *p* = .058, trend toward significance in longitudinal putamen volume and FA change between symp-HD and pre-HD; *p* = .057, trend toward significance in longitudinal putamen MD change between symp-HD and controls. | | | |
