# Supplementary material for: Multi-Modal Neuroimaging in Premanifest and Early Huntington’s Disease: 18 Month Longitudinal Data from the IMAGE-HD Study
Source: PLoS One. 2013 Sep 16;8(9):e74131. doi: 10.1371/journal.pone.0074131 (PMC3774648; doi:10.1371/journal.pone.0074131)
Supplement: Table S3 — Associations between MR percent change measures and clinical scores. (DOCX) [file pone.0074131.s006.docx]

| **Table S3. Associations between MR percent change measures and clinical scores.** | | | | | |
| --- | --- | --- | --- | --- | --- |
| Brain-wide | DBS | HD progression | Pre-HD progression | Symp-HD progression | UHDRS TMS |
| *Volume* |  |  |  |  |  |
| WB | -.27 | -.30^*^ | -.23 | .02 | -.15 |
| GM | -.21 | -.22 | -.25 | .03 | -.06 |
| WM | -.16 | -.20 | .01 | -.01 | -.16 |
| CSF | .17 | .07 | .05 | .03 | -.01 |
| Subcortical ROIs |  |  |  |  |  |
| *Volume* |  |  |  |  |  |
| Caudate | -.46*** | -.33** | -.20 | .12 | -.28* |
| Putamen | -.10 | -.25* | -.08 | -.10 | -.19 |
| *MD* |  |  |  |  |  |
| Caudate | .14 | .10 | .06 | -.25 | .003 |
| Putamen | .48^***^ | .35^**^ | .43^**^ | -.19 | .32^**^ |
| *FA* |  |  |  |  |  |
| Caudate | .08 | .22 | -.18 | .10 | .12 |
| Putamen | -.12 | -.10 | -.24 | .29 | .13 |
| Data are partial correlation coefficients and significance (superscript): ^*^ *p ≤ .*05; ^**^ *p ≤ .*01; ^***^ *p ≤ .*001. DBS, disease burden score; UHDRS TMS, Unified Huntington’s Disease Rating Scale, total motor score. All clinical measures are at baseline. Correlations with DBS were adjusted for age; correlations with UHDRS were adjusted for DBS and age; HD progression, pre-HD progression and sym-HD progression were controlled for CAG length. | | | | | |
