# Supplementary material for: Multi-Modal Neuroimaging in Premanifest and Early Huntington’s Disease: 18 Month Longitudinal Data from the IMAGE-HD Study
Source: PLoS One. 2013 Sep 16;8(9):e74131. doi: 10.1371/journal.pone.0074131 (PMC3774648; doi:10.1371/journal.pone.0074131)
Supplement: Table S4 — Annualized rates of change across volume, FA and MD measures. (DOCX) [file pone.0074131.s007.docx]

| **Table S4. Annualized rates of change across volume, FA and MD measures** | | | |
| --- | --- | --- | --- |
| Volume | Controls | Pre-HD | Symp-HD |
| Whole brain | 0.10 | -0.58 | -0.99 |
| Grey Matter | -0.05 | -0.83 | -1.08 |
| WM | 0.31 | -0.27 | -0.86 |
| CSF | 1.19 | 1.38 | 1.66 |
| Caudate | 0.25 | -1.04 | -2.09 |
| Putamen | 0.00 | -0.29 | -1.02 |
| MD |  |  |  |
| Caudate | 1.31 | 1.57 | 2.37 |
| Putamen | -1.01 | -1.21 | -0.05 |
| FA |  |  |  |
| Caudate | 0.82 | 1.61 | 3.56 |
| Putamen | 1.28 | 2.44 | 2.06 |
| Data are annualized rates of change for pre-HD*_far_*, pre-HD*_close_*, pre-HD*_all_*, symp-HD and age-matched controls. | | | |
